# An analysis of the trends, characteristics, scope, and performance of the Zimbabwean pharmacovigilance reporting scheme

**DOI:** 10.1002/prp2.657

**Published:** 2020-09-15

**Authors:** Josiah Tatenda Masuka, Star Khoza

**Affiliations:** ^1^ Harare Central Hospital Southerton, Harare Zimbabwe; ^2^ Department of Dermatology Nelson R Mandela School of Medicine University of KwaZulu‐Natal Congella, Durban South Africa; ^3^ Discipline of Pharmacology and Clinical Pharmacy School of Pharmacy Faculty of Natural Sciences University of the Western Cape Bellville South Africa

**Keywords:** adverse drug reaction, drug safety, pharmacovigilance, post‐market surveillance, spontaneous reporting system

## Abstract

We aimed to determine the reporting trends and characteristics of Individual Case Safety Reports (ICSRs) from the Zimbabwean national pharmacovigilance system. ICSRs submitted to VigiBase^TM^, the World Health Organisation's ICSR database between January 1993 and December 2017 were retrospectively reviewed with respect to the suspected medicine, System Organ Class (SOC), adverse drug reaction (ADR) type and seriousness, Anatomic Therapeutic Chemical (ATC) group, age, and gender. In total, 4071 ICSRs were submitted to VigiBase^TM^ from targeted spontaneous reporting (n = 2909; 71.5%), vaccine surveillance (n = 679; 16.7%), and passive spontaneous reporting (n = 483; 11.9%), respectively. The median age, ICSR completeness score and timeliness of reporting were 34.0 years (IQR: 14.0; 43.0), 0.90 (IQR: 0.70; 1.00), and 548.0 days (IQR: 266:1131), respectively. More than half of the ICRS were from female patients (n = 2233; 54.9%). Antiretrovirals, antibiotics, vaccines, and anti‐tubercular medicines were reported in 62.9%, 27.9%, 16.7%, and 13.3% of submitted ICSRs, respectively. The most frequent ADRs involved the skin and subcutaneous systems (n = 1111; 20.5%), nervous system (n = 733; 13.5%), and gastrointestinal disorders system (n = 654; 12.1%). The number of ADRs reported for each patient was significantly related to the reported medicine's ATC category (*P* = .001. The number of ADRs was significantly related to the use of antiretroviral agents. In conclusion, Zimbabwe has made significant progress in establishing a functional pharmacovigilance system. However, the present system reports on a limited therapeutic spectrum of medicines and potentially underestimates the national ADR burden. Further work is required to strengthen the more sustainable spontaneous reporting system which potentially captures a variety of therapeutic classes.

**Abbreviations**
ADR: adverse drug reaction
AEFI: adverse events following immunization
ATC: anatomic therapeutic class
ICH: International Conference on Harmonisation
ICSR: individual case safety report
MCAZ: Medicines Control Authority of Zimbabwe
MEdDRA: Medical Dictionary For Regulatory Activities
PIDM: programme for international drug monitoring
PMS: post‐marketing surveillance
PV: pharmacovigilance
SOC: system organ class
TSR: targeted spontaneous reporting
UMC: Uppsala Monitoring Centre
WHO: World Health Organisation

## 1 INTRODUCTION

The safety information on any medicinal product initially derives from pre‐clinical studies and randomized controlled trials during drug development. However, this information may not include all possible adverse drug reactions (ADRs) because of factors such as controlled clinical trial conditions and small sample sizes.^1^ Therefore, post‐marketing surveillance (PMS) provides important, additional safety information from millions of patients.^2^, ^3^ In light of this, the World Health Organisation (WHO) formed the Programme for International Drug Monitoring (PIDM) in 1968 to collect as many ADRs from diverse populations to best reflect and capture the aggregate safety profiles of individual medicines.^4^, ^5^ This global ADR data are contained in VigiBase^TM^, a WHO repository managed by the Uppsala Monitoring Centre (UMC).^5^ Analysis of VigiBase^TM^ data allows for early detection of serious and/or unexpected ADRs in addition to drug risk‐benefit analyses.^2^

The ADR burden in most Sub‐Saharan Africa (SSA) remains unknown despite training and analytical support from the UMC.^6^, ^7^ Developed countries contribute the majority of individual case safety reports (ICSRs) in VigiBase^TM^,^4^ whilst Africa contributes a mere 0.88% of the cumulative global ADR data.^8^ Most of these ADRs are related to antibiotics in contrast to the rest of the world where cardiovascular and neurological medicine related ICSRs predominate.^3^, ^8^ These observations reflect the unevenly distributed global disease burden, different drug utilization patterns, cultural norms, and medical practices.^2^ Furthermore, limited resources, government support and the over‐reliance on donor funded public health programmes may explain the preponderance for antibiotic related ICSRs in Africa.^9^ Moreover 50% of SSA countries lack the legal mandate to monitor ADRs and/or engage market authorization holders in PMS activities.^8^, ^9^ Consequently, the ADR profile of some medicines is inadequately reflected in their summary of product characteristics.

Zimbabwe previously boasted one of Africa's most robust healthcare systems, but over the past few years it has been stagnating due to decreasing government healthcare expenditure.^10^, ^11^ HIV/AIDS and tuberculosis have also contributed to the strain on resources necessitating donor funding for service provision.^10^, ^11^ However, pharmacovigilance activities for HIV and tuberculosis medicines have disproportionately been supported from donor funded public health programmes for the respective diseases.^12^ Non‐communicable diseases such as diabetes mellitus and hypertension have been steadily increasing^13^ and are projected to increase markedly in people living with HIV.^14^ Due to limited pharmacovigilance funding, the majority of any collated ADRs are expected to be from the mandatorily reported ICSRs linked to the funded programmes.

Zimbabwe's national pharmacovigilance scheme was established in 1993, but the country officially joined the PIDM in 1997.^8^ It currently utilizes passive spontaneous ADR reporting, vaccine safety surveillance and targeted spontaneous reporting (TSR) to collect unsolicited ADRs, adverse events following immunization (AEFIs) and anti‐retroviral and/or anti‐tubercular medicine related ADRs respectively. Stimulated reporting through TSR started in 2012^15^ after its promulgation by the WHO as a simple, inexpensive PV tool to leverage existing public health and PV programmes.^16^ TSR aimed to increase ICSRs by mentoring practitioners in high case‐load clinics whilst task shifting ADR reporting to non‐physician healthcare practitioners.^16^ All collected ICSRs are verified and collated centrally by the Medicines Control Authority of Zimbabwe (MCAZ), the national medicines regulator^17^ before causality assessment and subsequent upload into VigiBase^TM^.

Given the importance of continuous ADR data analysis,^18^ we set out to evaluate the trends and characteristics of Zimbabwean derived ICSRs in VigiBase^TM^. We also aimed to compare the reporting patterns for AEFIs and ADRs across all therapeutic areas; and to determine the relationship between age, gender, drug anatomic therapeutic class (ATC), reporter type and the number of reported ADRs.

## 2 MATERIALS AND METHODS

### 2.1 Study design

A retrospective descriptive analysis of anonymized ICSR data from Zimbabwe collected during the period 1 January 1993 to 31 December 2017 was conducted. The ICSR data was extracted from VigiBase^TM^ using VigiLyze®, the database's search and analysis software tool on 2018‐10‐06 (dataset date: 2018‐09‐30). We included ICSRs meeting the minimum criteria for regulatory reporting in accordance with the International Conference on Harmonisation of Technical Requirements for Registration of Pharmaceuticals for Human Use (ICH) E2D guideline.^19^

### 2.2 Classification of ICSRs

Adverse drug reaction preferred terms (PTs) were classified according to the Medical Dictionary for Regulatory Activities (MedDRA) version 21.0 System Organ Class (SOC), whereas the ICHE2A guideline's definitions were used to define serious ADRs.^20^, ^21^ All PTs were mapped to the corresponding primary SOC using the Bioportal MedDRA ontology repository.^22^ Only the main SOC affected was coded for each ICSR. The information about the pharmacological subgroup was classified according to the Anatomical Therapeutic Chemical (ATC) Classification system at level 2 for the suspected medicines.^23^ In this system, drugs are divided into five different levels based on the system or organ on which they act; their chemical, pharmacological and therapeutic properties.^24^ These classification criteria are similar to those used by Ozcan et al and de Vries et al^24^, ^25^

### 2.3 Statistical analysis

The de‐duplicated, MedDRA version 21.0 coded ADR data were exported into a Microsoft Office Excel^TM^ package for further analysis (Microsoft Corporation, Redmond, WA, USA). The timeliness of reporting was calculated by subtracting the date of onset of ADR from the date of VigiBase^TM^ entry.^26^ Where the date for the onset of the ADR was incomplete, the first day of the month was used, otherwise the ICSR was excluded from timeliness calculations.^26^ VigiGrade^TM^ completeness scores for ICSRs were obtained from VigiLyze® measuring the completeness of time‐to‐onset, age, gender, indication, outcome, report type, dose, country, primary reporter, and comments.^27^

Descriptive statistical methods were used to analyse the surveillance data. We used the ANOVA to compare continuous variables and the chi‐square test or Fischer's exact tests were used for categorical variables, as appropriate. Poisson regression analysis was done using the number of ADRs as the dependent variable and age, gender, ATC drug therapeutic class, number of drugs, and reporter type as independent variables. The Statistical Package for Social Sciences (SPSS) version 22.0 (IBM Corporation, Somers, NY, USA) and Stata 12 (StataCorp LLC, College Station, TX, USA) were used for the statistical analyses and for graphing the analyses respectively. All statistical tests were done at the 5% significance level.

### 2.4 Ethical considerations

Ethical exemption for the study was granted by the Medical Research Council of Zimbabwe (MRCZ Ref: MRCZ/E/207). The exemption was granted because ICSR data collection is a routine surveillance programme which uses anonymized data.

## 3 RESULTS

### 3.1 Characteristics of the pharmacovigilance system

**FIGURE 1:**
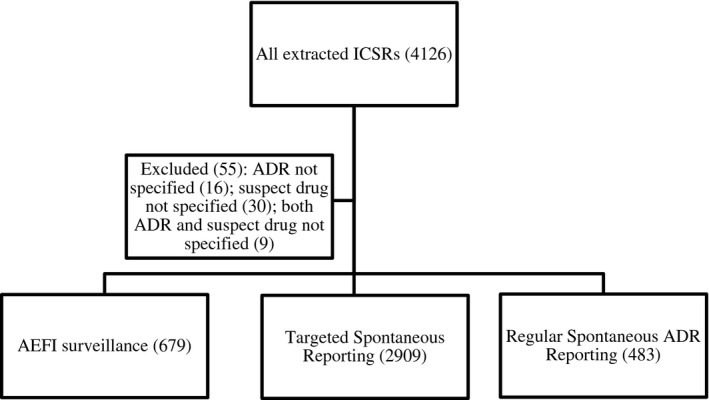
Schematic showing the overall distribution of all ICSRs extracted from VigiBase®

A total of 4126 ICSRs were extracted from VigiBase^TM^ for the period under review. Of these, 4071 ICSRs met the inclusion criteria and the average number of ADRs per ICSR was 1.33 ± 0.70 and the median number of ADRs^28^ per ICSR was 1.0 (range: 1‐7). The median time between the date of ADR occurrence and the date of ICSR entry in VigiBase^TM^ was 548.0 days (IQR: 266.0‐1131.0). The median VigiGrade^TM^ completeness score was 0.90 (IQR: 0.70‐1.00), indicating well‐documented ICSRs according to Bergvall et al^27^ The majority of the ICSRs were collected through the TSR programme (n = 2909; 71.5%) and the vaccine safety surveillance programme (n = 679; 16.7%) as shown in Figure 1. Passive spontaneous ADR reporting contributed 483 (11.9%) ICSRs. Nurses (n = 2767; 68.0%) reported the highest number of ICSRs, followed by medical doctors (n = 989; 24.3%), pharmacists (n = 195; 4.8%), and consumers (n = 5; 0.1%).

### 3.2 Characteristics of the ICSRs

#### 3.2.1 Demographic characteristics

The demographic, reporter and reporting characteristics are shown in Table 1. A total of 2233 (54.9%) of the ICSRs were recorded for females and 1792 (44.0%) were for males indicating an overall 0.80 male:female ratio. The male:female ratio was greater than 1 in children below 5 years of age (1.18) and infants (1.09). The male to female ratio was highest in children equal to or under 1 year of age, 1.75 (42 male:24 female ICSRs). The median age was 34.0 years (IQR: 14.0; 43.0). The majority of the ICSRs were from adults (n = 2880; 68.7%) while pediatric patients (≤16 years) comprised (n = 1069; 26.2%). However, most of the ICSRs were observed in the 20 ‐ 65 years age group (n = 2730; 67.0%), while 72 (1.8%) in adults older than 65 years of age.

**TABLE 1:** Demographic, reporter, and reporting characteristics

Characteristic	Total (N = 4071)	Vaccine (n = 679)	TSR (n = 2909)	Passive spontaneous reporting (n = 483)	*P*‐value
Age/years (mean ± SD)	30.90 ± 18.43	4.61 ± 5.83	37.16 ± 13.97	30.15 ± 20.99	<.001
Gender (M:F ratio)	0.80	1.10	0.76	0.73	<.001
ADR/ICSR ratio	1.33	1.52	1.26	1.52	^–^
Timeliness/days (mean ± SD)	857.47 ± 855.56	1180.86 ± 740.37	810.91 ± 859.84	683.27 ± 870.94	<.001
Completeness (mean ± SD)	0.81 ± 0.20	0.78 ± 0.15	0.84 ± 0.20	0.71 ± 0.22	<.001
Reporter type					
Nurse	2767 (68.0%)	609	1911	247	<.001
Pharmacist	195 (4.8%)	9	132	54	
Physician	989 (24.3%)	42	817	130	
Consumer	5 (0.1%)	0	1	4	
Not specified	115 (2.8%)	19	48	48	

#### 3.2.2 Reporting trends

**FIGURE 2:**
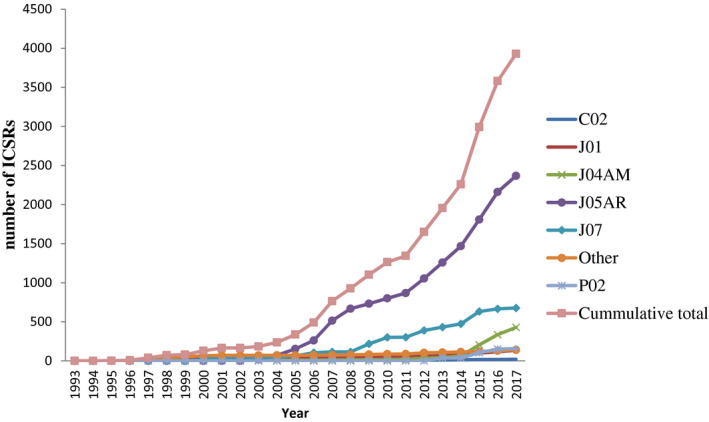
Year‐on‐year ICSR reporting trends

The annual number of ICSRs gradually increased during the study period. No reports were captured into VigiFlow® in the calendar years 1994, 1997, and 2002. However, a marked increase in the number of reports was noted starting in 2003. The increase in reporting of ICSRs was particularly notable for anti‐retroviral medicines (J05) and vaccines (J07) as shown in Figure 2.

### 3.3 Characteristics of the adverse drug reactions

The frequency of at least one serious ADR was 30.6% of the total ICSRs. Among serious ADRs, life threatening ADRs (n = 522; 12.8%) were the most frequent followed by significant disability/incapacitation (n = 378; 9.3%), and hospitalization (n = 194; 4.8%). In general, males tended to have more serious ADRs compared to females. This was particularly notable for deaths and incapacitation. The outcomes of the ADRs were generally poor, with 1981 (48.6%) of ICSRs recorded as either “not yet recovered,” 1787 (43.9%) or deaths 193 (4.7%). A total of 1156 (28.4%) were recorded as recovered whereas 935 ICSRs (23.0%) had no specified outcome (missing information). Children under one year had the highest death rate (14.2%; 56/395) followed by the 20‐65 year age group (3.5%; 83/2370).

The most frequent ADRs by SOC involved the skin and subcutaneous systems (n = 1111; 20.5%), nervous system (n = 733; 13.5%), and gastrointestinal disorders (n = 654; 12.1%) as shown in Table 2. The skin and subcutaneous tissue disorders also had the highest number of reported PTs per SOC followed by the nervous and gastrointestinal systems respectively. The 10 most commonly reported PTs were rash, gynaecomastia, peripheral neuropathy, vomiting, diarrhoea, dizziness, injection site abscess, lipodystrophy acquired, Steven Johnson syndrome and anaemia. Bivariate analysis indicated that frequency of patients who experienced more than two ADRs was higher among patients who received vaccines (9.1%), antimycobacterial agents (11.1%), and anthelminthic agents (13.5%) compared to patients who received antiretrovirals (5.2%), antihypertensive agents (8.0%), and antibacterial agents (8.2%). Poisson regression analysis indicated that the number of ADRs a patient experienced was associated with the therapeutic class (ATC code) of the prescribed medicine (*P* = .001) as shown in Table 3. Patient age (*P* = .858), gender (*P* = .362), reporter qualification (*P* = .093), and the number of drugs a patient was on (*P* = .539) were not related to the number of ADRs the patient experienced.

**TABLE 2:** Distribution of ADRs by system organ class

System organ class	Most commonly reported preferred term		SOC contribution to total PT count/ (n/%)
	Preferred term (PT)	PT contribution to SOC count/ (n/%)	
Blood and lymphatic system disorders	Anaemia	119 (47.0)	253 (4.7)
Cardiac disorders	Cardiomyopathy	12 (13.6)	88 (1.6)
Congenital, familial and genetic disorders	Talipes	6 (85.7)	7 (0.1)
Eye disorders	Eye inflammation	8 (14.6)	55 (1.0)
Gastrointestinal disorders	Vomiting	246 (37.6)	654 (12.1)
General disorders and administration site conditions	Pyrexia	135 (31.8)	424 (7.8)
Hepatobiliary disorders	Hepatitis	22 (31.9)	69 (1.3)
Immune system disorders	Stevens‐Johnson Syndrome	109 (38.3)	285 (5.3)
Infections and infestations	Pneumonia	20 (31.8)	63 (1.2)
Injury, poisoning and procedural complications	Injection site abscess	130 (51.0)	255 (4.7)
Metabolism and nutrition disorders	Hepatic enzyme increased	26 (10.0)	260 (4.8)
Musculoskeletal and connective tissue disorders	Pain in extremity		64 (1.2)
Neoplasms benign, malignant and unspecified (incl cysts and polyps)	Pathological fracture	6 (35.3)	17 (0.3)
Nervous system disorders	Neuropathy peripheral	298 (40.7)	733 (13.5)
Pregnancy, puerperium and perinatal conditions	Pregnancy	37 (53.6)	69 (1.3)
Psychiatric disorders	Psychotic disorder	17 (19.1)	89 (1.6)
Renal and urinary disorders	Renal impairment	42 (35.9)	117 (2.2)
Reproductive system and breast disorders	Gynaecomastia	418 (82.6)	506 (9.3)
Respiratory, thoracic and mediastinal disorders	Cough	30 (56.6)	53 (1.0)
Skin and subcutaneous tissue disorders	Rash	705 (63.5)	1111 (20.5)
Vascular disorders	Deep vein thrombosis	18 (26.5)	68 (1.3)

**TABLE 3:** Poisson regression analysis results for the number of reported ADRs/ICSR

Parameter	Hypothesis test			95% Wald CI for RR		
	Number of outcomes	Wald chi‐square	*P value*	Relative risk (RR)	Lower limit	Upper limit
Reporter qualification	3786	6.407	.093			
Reporter qualification = physician	960	.039	.844	1.078	.510	2.280
Reporter qualification = nurse	2648	.014	.907	1.046	.495	2.212
Reporter qualification = pharmacist	173	.290	.590	1.230	.578	2.617
Reporter qualification = consumer	5	.	.	1	.	.
Patient gender	3786	.833	.362			
Patient gender = female	2105	.833	.362	1.027	.970	1.087
Patient gender = male	1681	.	.	1	.	.
Number of recorded drugs	3786	8.930	.539			
Number of recorded drugs = 1	464	1.944	.163	.445	.143	1.389
Number of recorded drugs = 2	404	1.717	.190	.467	.149	1.459
Number of recorded drugs = 3	1385	1.911	.167	.448	.144	1.398
Number of recorded drugs = 4	978	1.994	.158	.441	.141	1.374
Number of recorded drugs = 5	300	1.902	.168	.449	.144	1.401
Number of recorded drugs = 6	113	1.554	.213	.483	.154	1.517
Number of recorded drugs = 7	63	2.159	.142	.420	.132	1.335
Number of recorded drugs = 8	61	1.662	.197	.468	.148	1.484
Number of recorded drugs = 9	15	1.410	.235	.480	.143	1.612
Number of recorded drugs = 10	2	.008	.929	1.065	.265	4.285
Number of recorded drugs = 12	1	.	.	1	.	.
ATC code	3786	23.044	.001			
ATC code = C02	23	.500	.479	.876	.606	1.265
ATC code = J01	124	3.669	.055	.812	.657	1.005
ATC code = J04AM	403	2.591	.107	.866	.727	1.032
ATC code = J05AR	2372	10.419	.001	.771	.658	.903
ATC code = J07	637	.448	.503	.941	.787	1.125
ATC code = P02	113	.149	.699	.956	.763	1.199
ATC code = others	114	.	.	1	.	.
Patient age	3786	.032	.858	1.000	.998	1.002

### 3.4 Characteristics of the drugs

**FIGURE 3:**
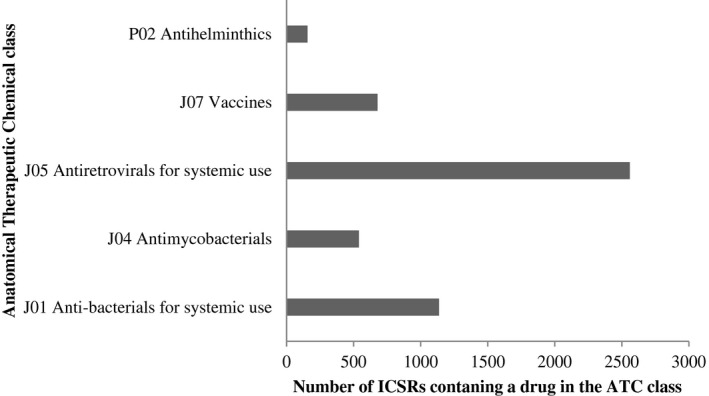
Reported Anatomical Therapeutic Chemical (ATC) classes

The median number of recorded drugs per ICSR was 3.00 (IQR: 3.00; 4.00). A total of 14 medicines were suspected in the majority (n = 4072; 86%) of ICSRs. The most frequently suspected medicines were efavirenz (n = 761; 18.7%), nevirapine (n = 447; 11.0%), isoniazid (n = 379; 9.3%), stavudine (n = 350; 8.6%), zidovudine (n = 300; 7.4%), and measles vaccines (n = 289; 7.1%). Over the study period, antiretrovirals, antibiotics, vaccines and anti‐tubercular medicines were reported in 2560 (62.9%), 1137 (27.9%), 679 (16.7%), and 540 (13.3%) of submitted ICSRs, respectively, as indicated in Figure 3. The most common ATC class in children below 5 years of age were vaccines while the proportion of anti‐retrovirals increased with patient age. ICSRs had a higher representation amongst females especially for anti‐retroviral and anti‐mycobacterial ATC classes compared to males who had a higher representation in the vaccine ATC class.

## 4 DISCUSSION

Our analysis shows that the total number of collected ICSRs has been steadily increasing in line with African and international trends.^4^, ^8^, ^29^, ^30^ The sharp increase in the number of ICSRs in 2004 may be explained by the increase in the number of public health programmes supported by development partners. However, marked increases in ICSRs mainly followed the introduction of active surveillance programmes in Zimbabwe. The cohort event monitoring (CEM) of artemisinin‐based combination therapies (ACTs) surveillance programme was introduced in 2008 and ended in 2010 while the active surveillance programme for the influenza A (H1N1) vaccine was implemented between 2009 and 2012. However, the CEM data was not included in this study because it was captured into CemFlow® and therefore was not available in VigiBase.^28^ In addition, Zimbabwe introduced TSR of selected essential medicines, including antiretrovirals and antitubercular drugs in 2012. The TSR active ADR surveillance programme partially explains the sharper increases in ICSR submissions in 2012. However, from in‐house experience, ICSR entry into VigiFlow depended on adequate staffing potentially explaining the observed ICSR trends. ICSRs were mainly submitted by healthcare workers in contrast to European trends where lawyers and the pharmaceutical industry contribute a significant proportion to the total number of submitted ICSRs.^29^ The latter has mostly been driven by strict legal reporting requirements which are not present in most African nations.^25^, ^29^ The markedly high reporting by nurses possibly shows a success of the TSR programme because its main purpose was to task shift ICSR reporting to non‐physician cadres.^16^ In addition, the nurse driven ICSR submission reflects the greater dependency of healthcare delivery in district hospitals on nurses as observed for the TSR programme and in Togo.^15^, ^31^

The most frequently reported SOCs were skin and subcutaneous tissue disorders, gastrointestinal disorders, general disorders and administration site conditions, and the nervous system disorders. This is similar to the Nigerian, Togolese, Colombian, French, Turkish and overall global findings.^4^, ^25^, ^31^, ^32^, ^33^, ^34^ However, some differences are notable between the Zimbabwean and French PV schemes on the prominence of blood, lymphatic system, and immune system disorders in the latter. The observed differences may be due to the varied ADR reporting practices in addition to differences in the most administered ATC groups within these countries.^4^ Moreover most ICSRs were reported for medicines in the ATC group J with a significantly higher proportion of anti‐retrovirals as previously shown in other low income countries such as Togo and Nigeria.^4^, ^8^, ^31^, ^34^, ^35^ In contrast, cardiovascular and nervous system medications were more frequently reported in developed countries, such as France and Germany.^29^, ^32^ The ADRs in Colombia and Portugal were mostly attributed to anti‐infectives for systemic use followed by nervous and cardiovascular system medicines.^33^, ^36^

The quality of ICSR reporting as indicated by ICSR timeliness and completeness is comparable to the global average but lags behind some established European pharmacovigilance schemes. The median time between the date of onset of an ADR and the date of reporting to VigiBase^TM^ of 2.35 years is comparable to the global average of 2.40 years.^4^, ^26^ However, the median timeliness was much longer than the 73 days observed in France and the 330 days observed in Uganda.^32^, ^37^ Timeliness has been noted to differ depending on the reporter qualification, the administered ATC groups and the number of reported ADRs per ICSR.^32^, ^37^ Furthermore, the median ICSR completeness score of 0.80 is significantly higher than the 0.41 for VigiBase^TM^ as a whole, but comparable to the yearly averages observed in the Indian PV scheme.^27^, ^38^ Regular refresher ADR reporting trainings for healthcare practitioners in Zimbabwe may explain the observed differences.^38^ Deficiencies in the timeliness of ICSRs indicate some immaturity of the Zimbabwean PV scheme and an area in need of improvement compared to long established PV schemes of ICH member countries. This could potentially be remedied by strengthening the PV system through introduction of PV specific legislation enforcing ICSR reporting and decentralizing the national PV system like in France and Germany.^29^, ^32^ Decentralization could also increase the efficiency and transparency of the national PV scheme.^39^

The ATC code had an influence on the number of reported ADRs whilst patient age, gender and the number of prescribed medicines had no influence as previously observed elsewhere.^40^ The number of ADRs was significantly related to the use of antiretroviral agents. ICSRs were observed to be more frequent in females regardless of the male to female gender ratio in the population as observed in Nigeria, Israel, Italy and the USAs.^34^, ^35^, ^40^, ^41^, ^42^, ^43^ However, ICSRs on deaths and significant disability/incapacitation were more common in males as previously observed in Italy and Sweden.^41^, ^44^ In contrast to the general trend, the male to female ratio was higher in children under 5 years of age and even higher in infants as expected from previous studies.^41^ In addition, it was noted that ICSRs were more common in the 20‐65 year age group possibly due to multiple drug therapy secondary to anti‐retroviral and anti‐tubercular drugs co‐prescription.^34^, ^41^, ^45^ This is supported by similar observations in Nigeria where comparatively higher HIV prevalence was noted in this age group.^34^, ^35^ The overall predominance of female ICSRs could be due to underlying physiological differences, females’ higher medical‐care seeking behavior and use of more prescriptions compared to males.^46^, ^47^

Despite the notable growth in the Zimbabwean pharmacovigilance scheme, mandatory reporting by market authorization holders should be considered in order to increase ICSR reporting. In addition, decentralization of ICSR collection to provincial and district healthcare facilities and the utilization of mobile phone ADR reporting platforms can strengthen the collation and completeness of ADR data. The latter measures may reduce barriers to reporting and extend the reach and availability of ADR reporting platforms to all relevant stakeholders including patients as observed in Kenya.^48^, ^49^ Furthermore, it is critical to provide relevant feedback to healthcare practitioners to provide meaning and an appreciation of ICSR reporting.^30^ This could subsequently help stimulate ICSR reporting, thereby increasing the numbers and spectrum of the submitted ICSRs. It is important to regularly review the performance of the scheme as a basis for informing regulatory measures.^29^ The spectrum of ICSRs could also be increased by encouraging the submission of ADRs from other therapeutic areas beyond anti‐infectives.

The inherent limitations of a study based on spontaneous ADR reports include under‐reporting and the inability to calculate incidence rates because of the unavailability of exposure/denominator data.^32^, ^42^ While ICSR data is voluntarily submitted, stimulated reporting through TSR introduced bias especially regarding the observed ADR, ATC, and SOC profiles. The ICSRs were skewed towards anti‐retroviral drugs, anti‐tubercular medicines and vaccines as would be expected given the dominance of public health programmes targeting HIV, tuberculosis, and immunization. The observed profiles may also indicate the prescription patterns, prevalent diseases and acute, severe and/or well‐known ADRs.^50^ The major strength of the current study is in highlighting areas in need of improvement and how the current set‐up compares to international regulatory pharmacovigilance schemes.

In conclusion, significant progress has been made in establishing a functional pharmacovigilance system. However, the present pharmacovigilance scheme is based on a limited therapeutic spectrum of medicines and may underestimate the ADR burden. In addition, there is need to improve the timeliness of ICSRs to enable further case reviews and timely signal detection.

## CONFLICT OF INTERESTS

There are no competing interests to declare.

## AUTHOR CONTRIBUTIONS

JM conceived and designed the study and contributed to data collection, data analysis, and interpretation of the results. SK contributed to data analysis and interpretation. Both authors reviewed the manuscript and approved the final version the final version of the manuscript.

## 5 DATA SHARING AND ACCESSIBILITY

The data that support the findings of this study are available from the corresponding author upon reasonable request.

## Supporting information

Fig S1‐S4

## References

[1] Stricker B , Psaty B . Detection, verification, and quantification of adverse drug reactions. BMJ. 2004;329(7456):44‐47. 15231627 10.1136/bmj.329.7456.44 PMC443457

[2] World Health Organisation . The safety of medicines in public health programmes: pharmacovigilance an essential tool. 2006 http://www.who.int/medicines/areas/quality_safety/safety_efficacy/Pharmacovigilance_B.pdf?ua=1. Accessed June 30, 2018

[3] World Health Organisation . Safety of medicines: a guide to detecting and reporting adverse drug reactions. Why health professionals need to take action. 2002; http://apps.who.int/iris/bitstream/handle/10665/67378/WHO_EDM_QSM_2002.2.pdf;jsessionid=051323D24D20EAEE9206E961AB1C8159?sequence=1. Accessed June 30, 2018

[4] Aagaard L , Strandell J , Melskens L , Petersen P , Holme HE . Global patterns of adverse drug reactions over a decade: analyses of spontaneous reports to VigiBase. Drug Saf. 2012;35(12):1171‐1182. 23072620 10.1007/BF03262002

[5] Lindquest M . VigiBase, the WHO global ICSR database system: basic facts. Drug Inf J. 2008;42(5):409‐419.

[6] Uppsala Monitoring Centre . Resources and support. 2017; https://www.who‐umc.org/global‐pharmacovigilance/who‐programme/resources‐and‐support/. Accessed June 30, 2018

[7] Olsson S , Pal S , Dodoo A . Pharmacovigilance in resource‐limited countries. Expert Rev Clin Pharmacol. 2015;8(4):449‐460. 26041035 10.1586/17512433.2015.1053391

[8] Ampadu HH , Hoekman J , de Bruin ML , et al. Adverse drug reaction reporting in Africa and a comparison of individual case safety report characteristics between Africa and the rest of the world: analyses of spontaneous reports in VigiBase(R). Drug Saf. 2016;39(4):335‐345. 26754924 10.1007/s40264-015-0387-4 PMC4796322

[9] Ampadu H , Hoekman J , Arhinful D , Amoama‐Dapaah M , Leufkens H , Dodoo A . Organizational capacities of national pharmacovigilance centres in Africa: assessment of resource elements associated with successful and unsuccessful pharmacovigilance experiences. Global Health. 2018;14(1):109. 30445979 10.1186/s12992-018-0431-0 PMC6240224

[10] Kidia K . The future of health in Zimbabwe. Glob Health Action. 2018;11(1):1496888. 30058477 10.1080/16549716.2018.1496888 PMC6070968

[11] Green A . Zimbawe post‐Mugabe era: reconstructing a health system. Lancet. 2018;391(10115):17‐18. 29323642 10.1016/S0140-6736(18)30007-2

[12] Olsson S , Pal SN , Stergachis A , Couper M . Pharmacovigilance activities in 55 low ‐ and middle‐income countries: a questionnaire based analysis. Drug Saf. 2010;33(8):689‐703. 20635827 10.2165/11536390-000000000-00000

[13] Mufunda J , Chatora R , Ndambakuwa Y , et al. Prevalence of noncommunicable diseases in Zimbabwe: results from analysis of data from the National Central Registry and Urban Survey. Ethn Dis. 2006;16(3):718‐722. 16937610

[14] Smit M , Olney J , Ford NP , et al. The growing burden of noncommunicable disease among persons living with HIV in Zimbabwe. AIDS. 2018;32(6):773‐782. 29369158 10.1097/QAD.0000000000001754 PMC5856639

[15] Masuka J , Chipangura P , Nyambayo P , Stergachis A , Khoza S . A Comparison of adverse drug reaction profiles in patients on antiretroviral and antitubercular treatment in Zimbabwe. Clin Drug Investig. 2018;38(1):9‐17. 10.1007/s40261-017-0579-z 28965312

[16] Pal S , Duncombe C , Falzon D , Olsson S . WHO strategy for collecting safety data in public health programmes: complementing spontaneous reporting systems. Drug Saf. 2013;36(2):75‐81. 23329541 10.1007/s40264-012-0014-6 PMC3568200

[17] Medicines Control Authority of Zimbabwe . Pharmacovilance centre. 2016; http://www.mcaz.co.zw/index.php/how‐we‐regulate/pharmacovigilance/pharmacovigilance‐centre. Accessed June 30, 2018

[18] Duijnhoven R , Straus S , Raine J , de Boer A , Hoes A , De Bruin M . Number of patients studied prior to approval of new medicines: a database analysis. PLoS Med. 2013;10(3):e1001407. 23526887 10.1371/journal.pmed.1001407 PMC3601954

[19] ICH . Post‐approval safety data management: definitions and standards for expedited reporting E2D. 2003; https://www.ich.org/fileadmin/Public_Web_Site/ICH_Products/Guidelines/Efficacy/E2D/Step4/E2D_Guideline.pdf. Accessed June 30, 2018

[20] ICH . Clinical safety data management: definitions and standards for expedited reporting E2A. 1994; https://www.ich.org/fileadmin/Public_Web_Site/ICH_Products/Guidelines/Efficacy/E2A/Step4/E2A_Guideline.pdf. Accessed June 30, 2018

[21] ICH . Introductory Guide MedDRA Version 21.0. 2018; 21: https://www.meddra.org/sites/default/files/guidance/file/intguide_21_0_english.pdf. Accessed October 07, 2018.

[22] Bioportal . Medical dictionary for regulatory activities. 2018; http://bioportal.bioontology.org/ontologies/MEDDRA?p=summary. Accessed October 14, 2018

[23] WHO Collaborating Centre for Drug Statistics Methodology. Guidelines for ATC classification and DDD assignment 2018 Collaborating Centre for Drug Statistics Methodology and the Nordic Council on Medicines, 21st edn Oslo, Norway WHO; 2017.

[24] de Vries S , Denig P , Ekhart C , et al. Sex differences in adverse drug reactions reported to the National Pharmacovigilance Centre in the Netherlands: an explorative observational study. Br J Clin Pharmacol. 2019;85(7):1507‐1515. 30941789 10.1111/bcp.13923 PMC6595313

[25] Ozcan G , Aykac E , Kasap Y , Nemutlu N , Sen E , Aydinkarahaliloglu N . Adverse drug reaction reporting pattern in Turkey: analysis of the national database in the context of the first pharmacovigilance legislation. Drugs ‐ Real World Outcomes. 2016;3(1):33‐43. 27747800 10.1007/s40801-015-0054-1 PMC4819489

[26] Letourneau M , Wells G , Walop W , Duclos P . Improving global monitoring of vaccine safety: a quantitative analysis of adverse event reports in the WHO Adverse Reactions Database. Vaccine. 2008;26(9):1185‐1194. 18243428 10.1016/j.vaccine.2007.12.033

[27] Bervall T , Noren G , Lindquist M . vigiGrade: a tool to identify well‐documentedindividual case reports and highlight systematic data quality issues. Drug Saf. 2014;37(1):65‐77. 24343765 10.1007/s40264-013-0131-x PMC6447519

[28] Suku CK , Hill G , Sabblah G , et al. Experiences and lessons from implementing cohort event monitoring programmes for antimalarials in four African Countries: results of a questionnaire‐based survey. Drug Saf. 2015;38(11):1115‐1126. 26267842 10.1007/s40264-015-0331-7 PMC4608977

[29] Dubrall D , Schmid M , Alesik E , Paeschke N , Stingl J , Sachs B . Frequent adverse drug reactions, and medication groups under suspicion: a descriptive analysis based on spontaneous reports to the German Federal Institute for Drugs and Medical Devices from 1978 to 2016. Dtsch Arztebl Int. 2018;115:393‐400. 29960607 10.3238/arztebl.2018.0393 PMC6041966

[30] Mehta U , Boulle A , Gouws J , et al. Pharmacovigilance: a public health priority for South Africa. SAHR. 2017. PMC5708547 29200789

[31] Potchoo Y , Yerima M , Gnandi TT , et al. Analysis of adverse reactions related to drugs and vaccines received at the national centre for pharmacovigilance from 2009 to 2016 in Togo. Pharmacol Pharm. 2018;9:344‐356.

[32] Thiessard F , Roux E , Miremont‐Salame G , et al. Trends in spontaneous adverse drug reaction reports to the French pharmacovigilance system (1986–2001). Drug Saf. 2005;28(8):731‐740. 16048358 10.2165/00002018-200528080-00007

[33] Machado‐Alba J , Londono‐Builes M , Echeverri‐Catano I , Ochoa‐Orozco SA . Adverse drug reactions in Colombian patients, 2007–2013: analysis of population databases. Biomedica. 2016;36:59‐66. 27622439 10.7705/biomedica.v36i1.2781

[34] Awodele O , Aliu R , Ali I , Oni Y , Adeyeye C . Patterns of adverse drug reaction signals in NAFDAC pharmacovigilance activities from January to June 2015: safety of drug use in Nigeria. Pharmacol Res Perspect. 2018;6(5), 10.1002/prp2.427 PMC6175912 30324768

[35] Ogar C , Abiola A , Yuah D , et al. A retrospective review of serious adverse drug reactions in the Nigerian Vigiflow database from September 2004 to December 2016. Pharmaceut Med. 2019;33(2):145‐157. 31933250 10.1007/s40290-019-00267-2

[36] Marques J , Ribeiro‐Vaz I , Pereira A , Polonia J . A survey of spontaneous reporting of adverse drug reactions in 10 years of activity in a pharmacovigilance centre in Portugal. Int J Pharm Pract. 2014;22(4):275‐282. 24188533 10.1111/ijpp.12078

[37] Kiguba R , Ndagije H , Nambasa V , Bird S . Adverse drug reaction onsets in Uganda's VigiBase: delayed international visibilty, data quality and illustrative signaldetection analyses. Pharmaceut Med. 2018;32(6):413‐427. 30546260 10.1007/s40290-018-0253-7 PMC6267548

[38] Kalaiselvan V , Kumar R , Thota P , Tripathi A , Singh G . Status of documentation grading and completenesss score for Indian individual case safety reports. Indian J Pharmacol. 2015;47(3):325‐327. 26069373 10.4103/0253-7613.157133 PMC4450561

[39] Mazzitello C , Esposito S , De Francesco AdeleE , Capuano A , Russo E , De Sarro G . Pharmacovigilance in Italy: an overview. J Pharmacol Pharmacotherap. 2013;4(Suppl 1):S20‐S28. 10.4103/0976-500X.120942 PMC3853663 24347976

[40] Zopf Y , Rabe C , Neubert A , et al. Women encounter ADRs more often than do men. Eur J Clin Pharmacol. 2008;64:999‐1004. 18604529 10.1007/s00228-008-0494-6

[41] Castellana E , Chiapetta M , Cattel F . Gender differences and pharmacovigilance: analysis in the Italian population. Ita J Gender‐Specific Med. 2018;4(1):27‐33.

[42] Moore T , Cohen M , Furberg C . Serious adverse drug events reported to the Food and drug administration, 1998–2005. Arch Intern Med. 2007;167(16):1752‐1759. 17846394 10.1001/archinte.167.16.1752

[43] Evans R , Lloyd J , Stoddard G , Nebeker J , Samore M . Risk factors for adverse drug events: a 10‐year analysis. Ann Pharmacother. 2005;39(7–8):1161‐1168. 15897265 10.1345/aph.1E642

[44] Holm L , Ekman E , Jorsater BK . Influence of age, sex and seriousness on reporting of adverse drug reactions in Sweden. Pharmacoepidemiol Drug Saf. 2017;26(3):335‐343. 28071845 10.1002/pds.4155

[45] Singh H , Dulhani N , Kumar B , Singh P , Tewari P , Nayak K . A pharmacovigilance study in in medicine department of tertiary care hospital in Chhattisgarg (Jagdalpur), India. J Young Pharm. 2010;2(1):95‐100. 21331200 10.4103/0975-1483.62222 PMC3035895

[46] Tran C , Knowles S , Liu B , Shear N . Gender differences in adverse drug reactions. J Clin Pharmacol. 1998;38(11):1003‐1009. 9824780 10.1177/009127009803801103

[47] Davies E , Green C , Mottram D , Pirmohamed M . Adverse drug reactions in hospitals: a narrative review. Curr Drug Saf. 2007;2(1):79‐87. 18690953 10.2174/157488607779315507

[48] Rubin D , Pesyna C , Jakubczyk S , Liao C , Tung A . Introduction of a mobile adverse event reporting system is associated with participation in adverse event reporting. Am J Med Qual. 2019;34(1):30‐35. 29938520 10.1177/1062860618781920

[49] Montastruc F , Bagheri H , Lacroix I , et al. Adverse drug reaction reports received through the mobile App, VigiBip®: a comparison with classical methods of reporting. Drug Saf. 2018;41(5):511‐514. 29270770 10.1007/s40264-017-0630-2

[50] Shin YS , Lee Y‐W , Choi YH , et al. Spontaneous reporting of adverse drug events by Korean regional pharmacovigilance centers. Pharmacoepidemiol Drug Saf. 2009;18(10):910‐915. 19621345 10.1002/pds.1796

