# Supplementary material for: An analysis of the trends, characteristics, scope, and performance of the Zimbabwean pharmacovigilance reporting scheme
Source: Pharmacol Res Perspect. 2020 Sep 15;8(5):e00657. doi: 10.1002/prp2.657 (PMC7507368; doi:10.1002/prp2.657)
Supplement: Supplementary file 1 — Fig S1‐S4 [file PRP2-8-e00657-s001.docx]

**APPENDIX**

**Article Type:** Original Research Article

**Title: An analysis of the trends, characteristics, scope and performance of the Zimbabwean pharmacovigilance reporting scheme**

**Short Title: Spontaneous Adverse Drug Reaction Reporting characteristics in Zimbabwe**

**Authors:** Josiah Tatenda Masuka^1,2^ and Star Khoza^3^

Affiliations

^1^Harare Central Hospital, PO Box ST14, Southerton, Harare, Zimbabwe

^2^Department of Dermatology, Nelson R Mandela School of Medicine, University of KwaZulu-Natal, Private Bag X7, Congella, Durban , 4013, South Africa

^3^Discipline of Pharmacology and Clinical Pharmacy, School of Pharmacy, Faculty of Natural Sciences, University of the Western Cape, Private Bag X17, Bellville 7535, South Africa

*Corresponding author:*

Dr Josiah Tatenda Masuka, Department of Dermatology, Nelson R Mandela School of Medicine, University of KwaZulu-Natal, Private Bag X7, Congella, Durban , 4013, South Africa; Email address: josiahmasuka@gmail.com

**Suppl. Fig 1: 5 y**ear interval ICSR reporting trends

**Suppl. Fig 2:** Year-on-year ICSR reporting trends

**Suppl. Fig 3:** Age range versus outcome

**Suppl. Fig 4:** Spread of preferred terms around System Organ Class (SOC)
